# Isolation, Identification, and Molecular Genetic Characteristics of a Pathogenic Strain of *Streptococcus suis* Serotype 3

**DOI:** 10.3390/pathogens14020192

**Published:** 2025-02-14

**Authors:** Longbai Wang, Jingli Qiu, Bing He, Xuemin Wu, Qiuyong Chen, Quanxi Wang, Renjie Wu, Bohan Zheng, Lunjiang Zhou, Xiaohong Huang

**Affiliations:** 1 Fujian Key Laboratory of Traditional Chinese Veterinary Medicine and Animal Health, College of Animal Science, Fujian Agriculture and Forestry University, Fuzhou 350002, China; wanglongbai@163.com (L.W.); wqx608@126.com (Q.W.); zhengbohan@fafu.edu.cn (B.Z.); 2 Institute of Animal Husbandry and Veterinary Medicine, Fujian Academy of Agricultural Sciences, Fuzhou 350013, China; qiujingli2020@163.com (J.Q.); he_bing_faas@163.com (B.H.); wxm0593@163.com (X.W.); fjchenqiuyong@163.com (Q.C.); wurenjie1231@163.com (R.W.); 3 University Key Laboratory for Integrated Chinese Traditional and Western Veterinary Medicine and Animal Healthcare in Fujian Province, College of Animal Science, Fujian Agriculture and Forestry University, Fuzhou 350002, China

**Keywords:** *Streptococcus suis* serotype 3, virulence factors, pathogenic, sequence analysis

## Abstract

*Streptococcus suis* (*S. suis*) is considered as one of the most crucial bacterial pathogens that leads to serious economic losses to the swine industry. Different *S. suis* serotypes exhibit diverse characteristics in population structure and pathogenicity. Epidemiology data underscore the importance of *S. suis* serotype 3 (SS3). However, except for a few epidemiological information, limited study information is available on this serotype. Herein, a pathogenic SS3 (the *S. suis* strain YA) was isolated from infected piglets in clinical practice, and then whole genome sequencing and analysis, hemolytic activity, antimicrobial susceptibility, pathogenicity to mice and piglets were conducted. The results of the whole genome sequencing of the *S. suis* strain YA showed that the complete genome was 2,167,682 bp in length with a G + C content of 41.2% and exhibited a unique sequence type (ST1801). The result of phylogenetic tree showed that it was most closely related to strain DNC15 and 6407 (ST54) from Denmark. The *tet(W)* and *erm(B)* resistant genes were identified in the *S. suis* strain YA by inserting into rum locus, in accordance with the result of resistance to tetracyclines and macrolide-lincosamide-streptogramin antibiotics. Twenty-seven key virulence factors were detected in the *S. suis* strain YA, including *sly*, *ef* and *mrp*, which contribute to pathogenicity in mice and piglets, causing bleeding and congestion in multiple tissue organs especially in the brains. And the LD_50_ value for mice was 1.54 × 10^7^ CFU. Therefore, our research emphasizes the importance of understanding SS3, and provides valuable information for the scientific prevention and control of *S. suis*.

## 1. Introduction

*Streptococcus suis* (*S. suis*), one of the important members of the Gram-positive *Streptococci*, is globally prevalent in the swine industry [1]. It colonizes the upper respiratory tract of swine, more particularly the tonsils and nasal cavities, as well as the genital and digestive tracts. In production and breeding, even though the carrying rate of *S. suis* in swine reaches 80%, morbidity rarely exceeds 5%. However, more than half of the reported *S. suis* attacks in pigs are cases of poor hygiene and concurrent disease that eventually result in meningitis, septicemia, arthritis, endocarditis, and pneumonia. Additionally, *S. suis* can cause septicemia and meningitis in humans [2]. It is thus considered a severe hazard to swine and public health, especially to related farmers and practitioners [1,3,4].

*S. suis* exhibits significant serotype diversity, with classification primarily based on capsular polysaccharide antigenic variations. To date, 29 distinct serotypes (1–19, 21, 23–25, 27–31, and 1/2) have been identified and characterized. Recent advancements in molecular typing have further expanded the understanding of *S. suis* diversity through the identification of novel capsular gene clusters. These include the discovery of Chz serotypes and 33 new capsular gene cluster variants (NCL1-21a, 21b-32), highlighting the ongoing evolution and genetic complexity of this pathogen [5,6]. Among all these serotypes, the principal infections in swine and humans are caused by serotype 2, on which a multitude of research has been centered. For the past few years, the clinical incidence rate of *S. suis* serotype 3 (SS3), one of the important serotypes causing clinical diseases in swine, has escalated. Currently, global reports of SS3 infections in swine approximate 15% of all *S. suis* diseases in swine [7]. Lunha et al. investigated the serotype distribution and pathotypic characteristics of *S. suis* isolates obtained from slaughtered pigs in Thailand, pointing out that the most prevalent serotype was serotype 8 (7.98%), followed by 19 (7.56%), 29 (6.72%), 3 (5.88%), and 2 (5.04%) [8]. Furthermore, SS3 is the second most prevalent serotype in North America and Asia [5]. However, there has been limited research on this serotype and researching SS3 is of great significance.

Literature has shown that the pathogenicity of *S. suis* is not entirely associated with serogroups alone, indicating that virulence factors are also essential in determining pathogenicity [9]. The *S. suis* genome contains multiple virulence factor genes, including *sly*, *mrp*, *fbps* and *orf*, and research on these virulence factors could provide a foundation for further studies on the bacterial pathogenicity.

Antibacterial drugs, including fluoroquinolones, amide alcohols, sulfonamides, aminoglycosides, and β-lactam drugs, play an important role in the prevention and treatment of *S. suis* infections [10]. However, with the non-standard use of antibiotics during the breeding process, the resistance of *S. suis* to these drugs has been increasing year by year. Samples isolated from large-scale pig farms revealed extremely high resistance rates to various antibiotics [11], and these *S. suis* serve as a reservoir of antibiotic resistance genes and pose a great threat to public health. It is crucial to monitor the antimicrobial susceptibility of *S. suis* in pigs.

In the present study, suspected *S. suis* samples were isolated from a diseased pig farm in Fujian Province, China in 2022. The piglets were produced by a pig farm in China, through the introduction of breeding pigs from Denmark. The isolated strain underwent serotype determination via PCR, followed by a comprehensive analysis through whole genome sequencing (WGS), including verification of the presence of pertinent virulence genes and determination of the strain’s molecular genetic characteristics. Additionally, antimicrobial susceptibility, pathogenicity, and histopathological damage in pigs and mice were evaluated. This work contributes to the development of new strategies required for the control and treatment of *S. suis* infections in pigs and humans.

## 2. Materials and Methods

### 2.1. Isolation and Identification of S. suis Strain YA

The *S. suis* strain YA was isolated in 2022 from the lung of a nursery pig with neurological symptoms in Fujian Province, China. The piglets were produced by a pig farm in China through the introduction of breeding pigs from Denmark. The lungs and intestines of dead pigs, which tested negative for swine fever, porcine reproductive and respiratory syndrome virus, pseudorabies, and circovirus, were inoculated onto fresh sheep blood agar plates containing Nicotinamide Adenine Dinucleotide. After anaerobic cultivation at 37 °C for 24 h, a single suspicious colony was selected for purification. Then, the single pure cultured bacterial colony was observed with Gram staining and a microscope to determine the morphological characteristics of the bacteria.

Thereafter, the strain was validated as *S. suis* and serotyped as *Cps3L* with PCR analysis and sequencing. The 16S rRNA classification was performed according to a standard procedure using the universal primers: Pr1 5′-AGAGTTTGATCCTGGCTCAG-3′ and Pr2 5′-TACGGCTACCTTGTTACGACTT-3′. The 16S rRNA serotype identification was performed according to a standard procedure using the specific primers of *S. suis Cps3L*: Pr1 5′-ACATCCATTGCAGGAGTAGT-3′ and Pr2 5′-TGCAGTTCCAAAATTCTTCGT-3′.

### 2.2. Genome Sequencing and Analysis

The *S. suis* strain YA was further analyzed through WGS. The quality of the genomic DNA extraction was tested using a NanoDrop One spectrophotometer (NanoDrop Technologies, Wilmington, DE, USA) and a Qubit 3.0 Fluorometer (Life Technologies, Carlsbad, CA, USA). The whole genome of *S. suis* YA was sequenced using the Nanopore PromethION (Oxford Nanopore Technologies, Oxford, UK) and Illumina NovaSeq 6000 platforms (Illumina, San Diego, CA, USA). The Nanopore PromethION sequence reads were assembled with GUPPY (Version: 5.0.16), and corrected in Illumina NovaSeq 6000 with fastp (Version: 0.23.2). The antibiotic resistance gene analysis was performed using ResFinder 4.5 (http://genepi.food.dtu.dk/resfinder, accessed on 20 November 2024). The gene sequence predicted with these functional databases was compared to other similar stains using BLAST+ (Version: 2.11.0+). MinCED (Version: 0.4.2)was used to analyze the CRISPR element. The R package Circlize (Version3.12.0) was used to analyze the Circular map of the *S. suis* strain YA genome.

### 2.3. Bioinformatic Analysis

The complete genome sequences of 23 different strains of *S. suis* isolates were obtained from NCBI GenBank and used for phylogenetic analyses (Table 1). These sequences included the *S. suis* strain YA genome provided by our research group (CP149804.1). The dataset was aligned in KSNP4.1. Then, 106,379 SNPs were generated according to Kemer’s theorem [12]. A phylogenetic tree of the whole genome dataset was constructed using the IQ-TREE2.3.6 software based on the 106,379 SNPs loci using the maximum likelihood (ML) [13], and as an outgroup, *Streptococcus pneumoniae* ATCC 700669 (NC_011900) was used to root the tree. The best-fit model of evolution was determined as TVM + F+ASC based on BIC values, and 1000 repeated calculations were performed.

The MLST analysis of *S. suis* genomes was determined using the PubMLST database [14]. The distribution of 35 putative virulence-associated genes of *S. suis*, as described in previous reports [15], was investigated among the *S. suis* strain YA and other *S. suis* genomes (with coverage > 80% and a nucleotide sequence identity > 80%). The information of 35 putative virulence-associated genes analyzed in this study was listed in Table S2.

### 2.4. Hemolytic Activity

The *S. suis* strain YA was cultured overnight at 37 °C and centrifuged at 10,000 rpm for 10 min at 4 °C. Subsequently, the supernatant was collected and two-fold diluted with phosphate-buffered saline (PBS) (pH 7.4). Defibrated sheep blood was washed twice with PBS and 0.5 mL of 4% defibrated sheep blood was added to 0.5 mL of the supernatant. Then, the mixture was incubated in a water bath at 37 °C for 1 h and centrifuged at 1000 rpm for 10 min. Finally, 200 µL aliquots of supernatant were collected and measured at OD 540 nm. Additionally, a sample treated with 2.5% Triton X-100 was set as a reference for 100% lysis.

### 2.5. Antimicrobial Susceptibility Testing

The antibiotic susceptibility of the *S. suis* strain YA was tested with a disk diffusion method based on the Performance Standards for Antimicrobial Susceptibility Testing, according to the guidelines of the Clinical and Laboratory Standards Institute [16]. The following antimicrobial agents were used: penicillin (10 µg), ampicillin (10 µg), piperacillin (100 µg), cefuroxime sodium (30 µg), ceftazidime (30 µg), ceftriaxone (30 µg), cefoperazone (75 µg), amikacin (30 µg), gentamicin (10 µg), streptomycin (10 µg), minocycline (30 µg), erythromycin (15 µg), norfloxacin (10 µg), ciprofloxacin (5 µg), lincomycin (2 µg), vancomycin (30 µg), trimethoprim-sulfamethoxazole (25 µg), chloramphenicol (30 µg), clindamycin (2 µg), levofloxacin (5 µg), imipenem (10 µg), and doxycycline (30 µg), which were obtained from BKMAMLAB (Changsha, China). *Staphylococcus aureus* ATCC 29213 was served as the quality.

### 2.6. Pathogenicity of the S. suis Strain YA in Mice

Kunming mice were chosen as the animal model in this experiment and the abovementioned *S. suis* strain YA, PT1016, PTSF9, ND1111, ND1114 and ND0827 *S. suis* strains, which were stocked in our lab were cultured in TSA containing 5% (*v*/*v*) sheep blood for 16–20 h, and then washed twice with PBS. The background information on the strains was listed in Table S3. The washed *S. suis* was re-suspended in PBS to a concentration of 2 × 10^8^ CFU/mL and the suspension was intraperitoneally injected with a 0.2-mL syringe into 5 mice. Mice injected with PBS were set as the negative control. The mortality of the treated mice was monitored and recorded for several days post-infection.

### 2.7. LD_50_ Testing of the S. suis Strain YA

To test the virulence of the *S. suis* strain YA, the median lethal dose (LD_50_) was determined. Sixty Kunming mice were randomly assigned to six groups with 10 mice in each group. The resuscitated *S. suis* strain YA bacteria were washed with a PBS solution three times. Additionally, the bacterial concentrations were adjusted to 5.6 × 10^8^, 1.7 × 10^8^, 5.2 × 10^7^, 1.6 × 10^7^, and 4.8 × 10^6^ CFU and intraperitoneally injected with a 0.2-mL syringe into experimental mice from each group, respectively. Ten control mice were inoculated only with an equivalent volume of PBS solution. Mortality and clinical manifestations of the treated mice were monitored until 14 days post-infection. The LD_50_ value was calculated according to the computational formula below [17]:

(1) $${LD}_{50}={lg}^{-1}[X_{m}-i(\sum p-0.5)]$$

(2) $$S_{x50}=i\sqrt{\sum \frac{pq}{n}}$$

(3) $$CI\;of\;95\%={lg}^{-1}\;(lg{LD}_{50}\pm 1.96\times S_{x50})$$

where *X_m_* is the *LgLD*_50_, *i* is the group distance (the difference between the logarithmic values of adjacent dose groups), *p* is the mortality of each group of animals, *q* is the survival rate of each group of animals, *S_x_*_50_ is the standard error, and *n* is the number of animals in each group.

### 2.8. Pathogenicity of the S. suis Strain YA in Piglets

Ten 25-day-old piglets were randomly divided into two groups of five each, with one group designated as the control. The challenge group received a 0.5-mL nasal drip and a 1.5-mL intramuscular injection of the bacterial mix at a concentration of 2 × 10^7^ CFU/mL. The groups were observed and recordings were made after the challenge. The negative control group was inoculated with an equal amount of PBS using the same inoculation method.

### 2.9. Histological Analysis

Samples from the heart, liver, spleen, lungs, kidneys, and brain of mice as well as samples from the lymph nodes, liver, spleen, lungs, kidneys, and brain of piglets were immersed in 10% buffered formalin. After paraffin embedding, tissue sections were stained with H&E according to the standard protocol and examined under light microscopy.

## 3. Results

### 3.1. Isolation and Identification of the S. suis Strain YA

The *S. suis* strain YA was isolated from sick sows from a large-scale pig farm in Fujian Province. Piglets in the sow delivery room which were about to be weaned and piglets transferred to the nursery exhibited clinical features such as inappetence, fervescence, swollen joints and neurological symptoms, with an incidence of approximately 51%, and a mortality rate of about 30%. After anaerobic cultivation on sheep blood agar plates for 24 h, hemolytic colonies the size of a needle tip, gray-white and semi-transparent, with a smooth surface, circular shape, raised edge, and neat edge grew. The 16S rRNA and *cps 3L* gene fragments were amplified from the bacterial genome through the polymerase chain reaction (PCR). A subsequent agarose gel electrophoresis analysis of the PCR products revealed distinct bands at approximately 1500 (bp) and 340 bp, respectively (Figure S1). The result of 16S rRNA sequencing and serotype identification illustrated that the strain is *S. suis* serotype 3.

### 3.2. Features of Genome and Bioinformatic Analysis

**Figure 1:**
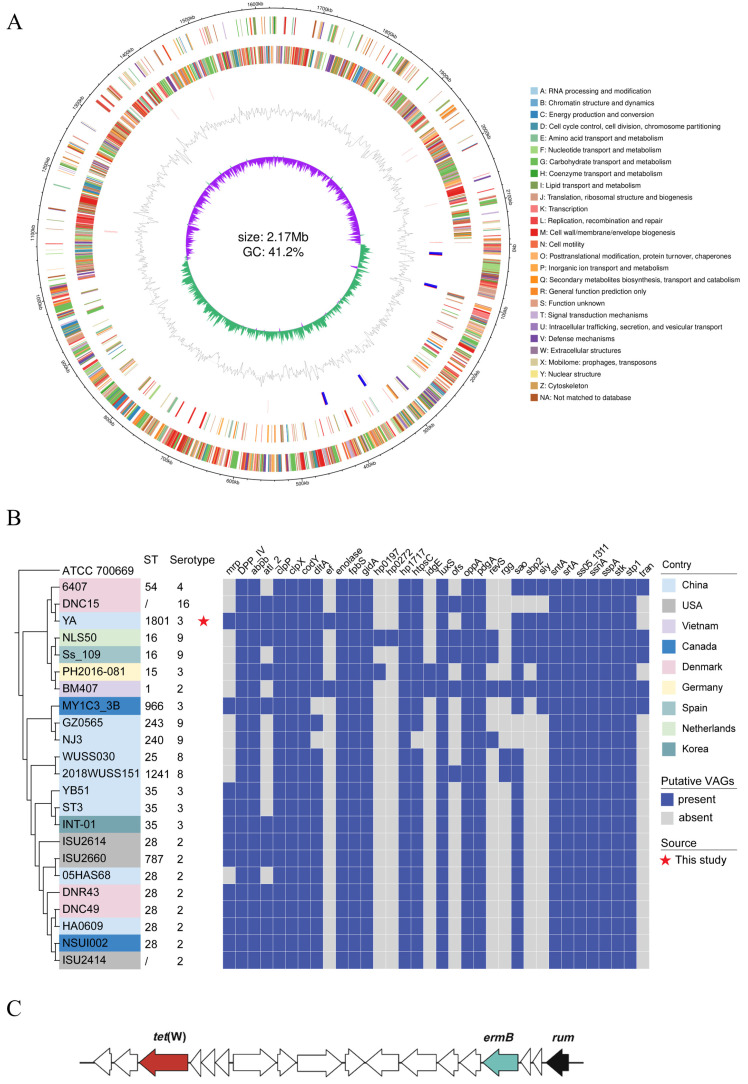
The *S. suis* strain YA genomic features and bioinformatic analysis. (**A**) Circular map of the *S. suis* YA genome generated using R package Circlize. (**B**) The phylogenetic tree and information of STs and putative virulence-associated genes (VAGs) for the *S. suis* serotype 3 strain YA and other serotype strains. The phylogenetic tree was constructed based on the SNPs of the core genome. The *S. pneumoniae* ATCC 700669 (NC_011900) was used as an outgroup to root the tree. (**C**) Genetic context of antibiotic resistance genes in *S. suis* serotype 3 strain YA.

The complete genome of the *S. suis* strain YA consisted of one chromosome (NCBI GenBank accession number CP149804) without any plasmid. The complete genome was 2,167,682 bp in length with a G + C content of 41.2%, containing 2171 genes and 2049 coding sequences (CDSs), comprising 56 tRNA genes (four of the 5S, 16S, and 23S rRNA genes each) (Figure 1A).

The MLST analysis revealed that the *S. suis* strain YA exhibited a unique sequence type (ST1801), which is notably distinct from other serotype 3 strains (Figure 1B). To investigate the phylogenetic relationships among the *S. suis* strains, we analyzed 23 complete genomes of different serotypes from the NCBI GenBank dataset. The ML phylogenetic tree (Figure 1B) showed that the different serotypes formed different evolutionary relationships, promoting biodiversity. The *S. suis* strain YA was most closely related to the DNC15 (serotype 16) sample from Denmark, as well as to the 6704 (serotype 4), which was also from Denmark. It is speculated that there are genetic correlations between the *S. suis* strain YA and these two strains.

The serotype 3 strains (INT-01, ST3, and YB51) were clustered together with eight serotype 2 strains in the phylogenetic tree, with the same ST (ST35), suggesting that these strains were probably derived from a recent common ancestor. Surprisingly, the *S. suis* strain YA and a few other SS3 strains (MY1C3 and PH2016-081) were distantly related to the other SS3 strains INT-01, ST3, and YB51.

A total of 35 putative virulence-associated genes that are predominantly found in highly pathogenic *S. suis* serotype 2 strains were analyzed in comparison to the *S. suis* strain YA and other serotype strains (Figure 1B). The *IdeSsuis* gene was not detected in all trains. As shown in the result, 27 of 35 putative virulence-associated genes were present in the *S. suis* strain YA. Compared to other serotype 3 strains, the *S. suis* strain YA carried a greater number of virulence-associated genes. It should be noted that the classical VAG profile (ef/*sly*/*mrp*) was found in the *S. suis* strain YA. Genes of *ef*, *mrp*, and *sly* are mostly associated with strains with high virulence, and it is interesting that those genes were all detected in the *S. suis* strain YA.

The *S. suis* strain YA contained six insertion sequence (IS) families, namely, ISL3, IS110, IS982, IS4, IS3, and IS30. The MinCED analysis showed one CRISPR element with a DR (direct-repeat) consensus sequence length of 36 bp (GTTTTGAAACCATTCGAAACAGCACAGCTCTAAAAC) in the *S. suis* strain YA.

ResFinder 4.5 identified *tet(W)* and *erm(B)*, which confers resistance to tetracycline and macrolide-lincosamide-streptogramin (MLSB). Several studies have demonstrated that *tet(O)* and *erm(B)* are commonly found in the *S. suis* strains isolated from pigs and humans worldwide, while *tet(W)* was found additionally in the strain YA. As shown in Figure 1C, the organization of the gene *tet(W)* and gene *erm(B)* was identical in the strain YA. It is worth mentioning that the MLSB resistance gene *erm(B)* and tetracyclines resistance gene *tet(W)* were detected within integrative strains by inserting into rum locus. There was no classic ICE found in the *S. suis* strain YA.

### 3.3. Hemolytic Activity

**Figure 2:**
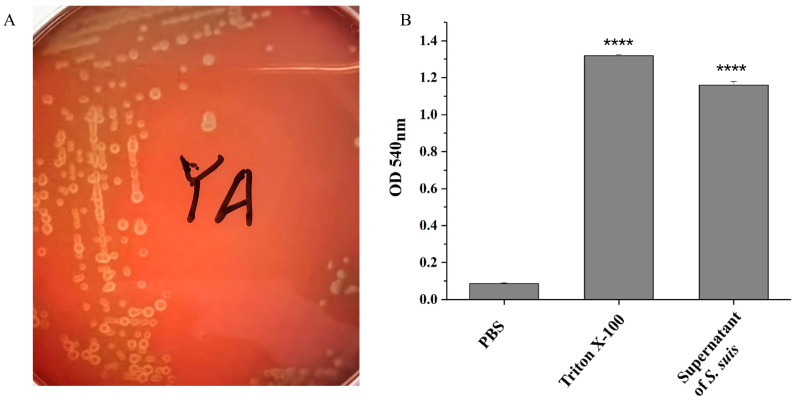
Hemolysis analysis of the *S. suis* strain YA. (**A**) Colony morphology of the *S. suis* strain YA on sheep blood agar plates. (**B**) The OD 540 nm value of PBS (negative control), Triton X-100 (positive control), and supernatant of *S. suis* culture. **** indicates a highly difference compared to the PBS group (*p* < 0.0001).

On sheep blood agar plates, the colonies of the *S. suis* strain YA exhibited distinct morphological characteristics, demonstrating a clear zone of hemolysis surrounding the bacterial growth. This hemolytic phenomenon, indicative of complete red blood cell lysis, was consistently observed around the colonies (Figure 2A). Additionally, we discovered that the supernatants of the *S. suis* strain YA culture medium showed hemolytic activity that is capable of lysing more than 85% of the defibrated sheep erythrocytes (Figure 2B).

### 3.4. Antimicrobial Susceptibility Testing

The results of the susceptibility tests showed that the *S. suis* strain YA is resistant to multiple antibiotics commonly used in clinics. Overall, the isolates displayed resistance to cefuroxime sodium, amikacin, gentamicin, streptomycin, minocycline, doxycycline, erythromycin, lincomycin, clindamycin, and trimethoprim-sulfamethoxazole. It was also moderately resistant to piperacillin, norfloxacin, and vancomycin and susceptible to penicillin, ampicillin, ceftazidime, ceftriaxone, cefoperazone, ciprofloxacin, chloramphenicol, levofloxacin, and imipenem.

### 3.5. Pathogenicity in Mice

**Figure 3:**
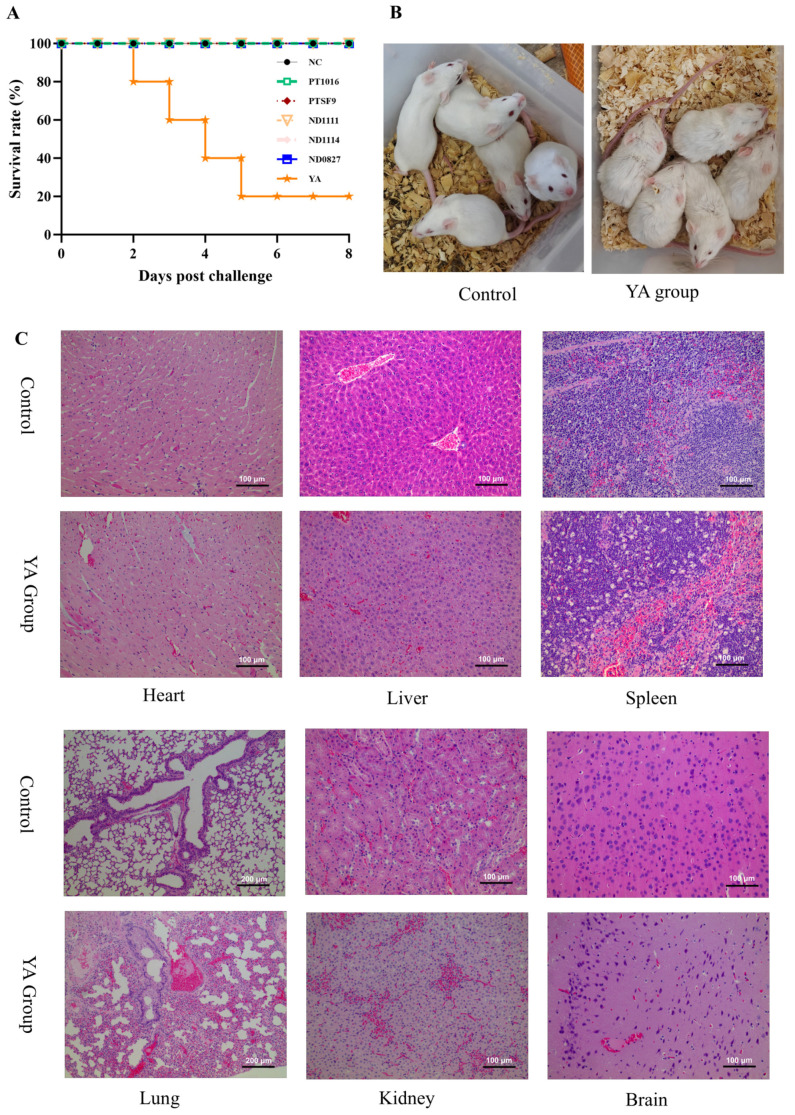
Pathogenicity of the *S. suis* strain YA in Kunming mice. (**A**) Survival rate of mice in each group after infection with different strains of *S. suis*. (**B**) Clinical symptoms of mice infected with *S. suis* strain YA with neurological symptoms, as well as rough and messy fur. (**C**) Histopathological changes in the heart, liver, spleen, lung, kidney, and brains of uninfected mice and mice infected with the *S. suis* strain YA.

The pathogenicity testing results of the *S. suis* strains in mice are shown in Figure 3A. There were no fatal cases with infection of strains PT1016, PTSF9, ND1111, ND1114, and ND0827. In contrast, the *S. suis* strain YA resulted in the death of one mouse within two days post-injection, with four mice dying in total within five days. As a consequence, the *S. suis* strain YA with the strongest virulence was selected from the above isolates for further study.

The LD_50_ value was determined to evaluate the pathogenesis of the *S. suis* strain YA. After 14 days of the *S. suis* strain YA infection in mice, the mortality rates of each group were calculated and are shown in Table S1. Based on the different initial doses of the *S. suis* strain YA given to Kunming mice, the LD_50_ value was determined to be 1.54 × 10^7^ CFU, suggesting that the *S. suis* strain YA is pathogenic [18]. There were no corresponding symptoms or deaths in the control group of mice.

Except for group V, the mice in each group rapidly exhibited symptoms, including multiple manifestations of mental depression, rough and messy fur, decreased appetite, diarrhea, fear of cold, huddling and neurological symptoms (Figure 3B). Notably, group I mice fell ill immediately and died within 12 h of infection. The number of deaths peaked between 12 and 24 h and then stabilized. Dissection of the dead mice revealed varying degrees of congestive lesions in multiple tissues and organs (Figure S2). Notably, congestion and swelling were found in the lungs and brain, suggesting that the *S. suis* strain YA can cause serious damage to these organs.

The H&E staining revealed histopathological changes, which validated the macroscopic lesions observed in clinical tissues (Figure 3C). Compared with the control group, the heart showed no significant changes in the challenged group, although the YA-infected mice presented with injuries in other tissues, manifesting as severe bleeding and congestion. Degeneration, necrosis, and nuclear lysis were seen in the liver cells and lymphocytes were absent in the spleen, which contributed to the exposure of the endothelial cells. The lung tissues suffered from thickening alveolar walls, leading to interstitial pneumonia. The *S. suis* strain YA induced significant bacterial encephalitis with an increase in microglia and inflammatory cells around the small blood vessels, and the presence of vascular proliferation and neuronal phagocytosis. Conclusively, the pathological changes in brain tissue correspond to and explain the clinical neurological symptoms.

### 3.6. Pathogenicity in Piglets

**Figure 4:**
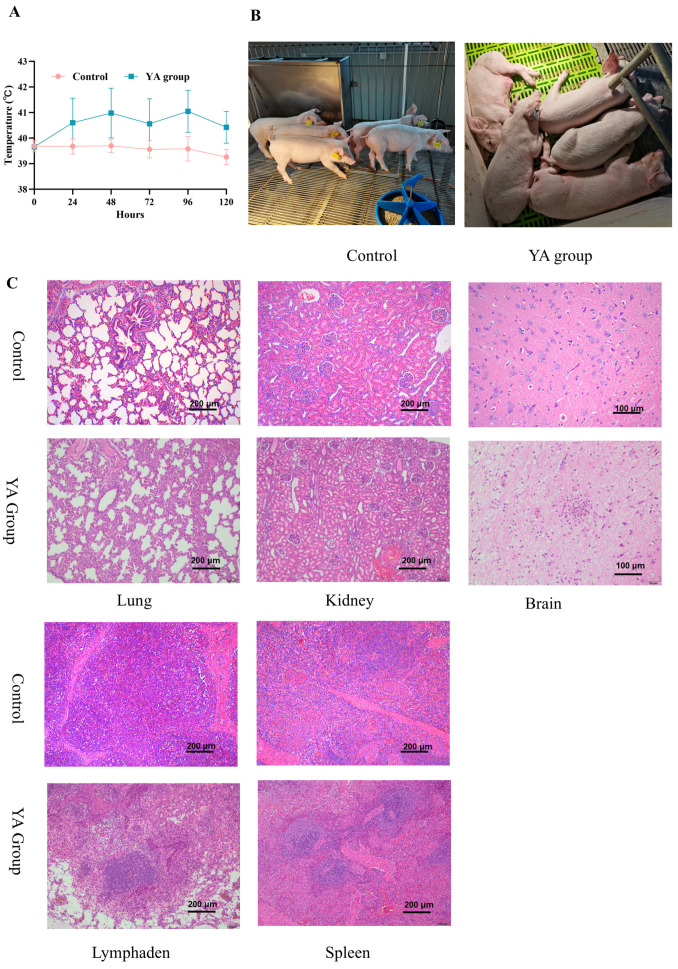
Pathogenicity of the *S. suis* strain YA in 25-day-old piglets. (**A**) Temperature changes in piglets after the challenge. (**B**) Clinically normal control group and fervescence, mental depression, hemiplegia and inability to get up with clinical manifestations in challenged piglets. (**C**) Histopathological changes in the lung, kidney, brains, lymphaden, and spleen of uninfected piglets and those infected with strain YA.

For the challenged group, the body temperature of the piglets increased significantly after infection (Figure 4A), reaching a maximum of 41.9 °C. Forty-eight hours after infection, the piglets showed symptoms of poor mental health and decreased appetite. By 72 h, the piglets developed clinical symptoms such as drooling, joint swelling, and even an inability to get up. One piglet in the challenged group died 96 h after infection, while the remainder had body temperatures of 41 °C. At 120 h after infection, symptoms such as watery limbs, the inability to get up, wheezing and convulsions appeared (Figure 4B). The negative control group had normal mental health and appetite, indicating that the *S. suis* strain YA is pathogenic.

After infection with the *S. suis* strain YA, microscopy showed obvious interstitial pneumonia and thickening of alveolar septa (Figure 4C). In the kidney, interstitial congestion and bleeding occurred and brain lesions were characterized by meningeal congestion, bleeding, infiltration of several inflammatory cells mainly comprising neutrophils, as well as the appearance of neuronal phagocytosis and vascular proliferation. The number of lymphocytes in the lymph nodes decreased, with the infiltration of many inflammatory cells, mainly comprising neutrophils. In addition, there was interstitial congestion, bleeding, and deposition of hemosiderin in the lymph nodes. In the spleen, the lesions were not severe, with local red pulp congestion and a small amount of inflammatory cell infiltration. No significant pathological changes were observed in the tissues of the negative control group. In summary, the pathological section results indicated that the *S. suis* strain YA caused lesions in multiple organs of piglets, especially in brain tissue and lymph nodes, which contributed to the development of neurological symptoms and immune deficiencies.

## 4. Discussion

*S. suis* is a significant bacterial pathogen that adversely affects the healthy development of the swine industry. It often occurs alone or as a secondary pathogen, causing serious economic losses to the swine farming industry. At present, research on *S. suis* mainly focuses on dominant serotypes (type 2), with relatively few reports on any other serotypes. However, in clinical practice, the other serotypes of *S. suis* (such as types 3 and 4) have been detected and isolated, while some strains are highly pathogenic [1,19,20,21,22]. Herein, six *S. suis* strains were isolated from pigs in Fujian Province, China. Most of these were non-virulent, illustrating that most pig farms carry *S. suis*, without clinical problems. The *S. suis* strain YA isolated from a newly built large-scale pig farm in Fujian Province in this study was identified as serotype 3, a serotype that has shown pathogenicity in pigs. All the breeding pigs in this farm were imported from Denmark, and piglets produced by sows began by joint enlargement and neurological symptoms. Infection was accompanied by a high incidence rate (about 51%), and a high mortality rate (more than 30%). Newborn piglets with hypoimmunity were the first to show symptoms. This suggests that SS3 is a serious threat to pigs and it is necessary to take this *S. suis* serotype seriously.

In recent years, the principal literature about SS3 was focused on epidemiological investigations. There are relatively few reported isolates of SS3, including ST3, INT-01, YB51, PH2016-081, and MY1C3-3B. However, there have been no reports involving the virulent SS3 strain. Whole genome sequencing and analysis on the clinically isolated virulent *S. suis* strain YA was performed in this study, and the virulence of the *S. suis* strain YA was validated in mice and piglets. With SS3 becoming a predominant serotype in epidemiological investigations, the results may provide important insights into the development of control strategies for clinical SS3 infection.

Whole genome sequencing revealed the basic characteristics of the bacterial genome, contributing to the exploration and study of the virulence and resistance genes, which is of great significance for investigating pathogenic mechanisms. Studies have reported that sequence changes in the *cps* gene could reflect the phylogenetic relationship between *S. suis* strains [23] and that *Cps3L* is a gene specific to SS3. In this study, the homology of the nucleotide sequences of the *Cps3L* gene from the isolated *S. suis* strain YA and the *S. suis Cps3L* gene (KC537365.1) registered in GenBank was up to 100%, while the homology with other serotype 3 *S. suis* strains in China and overseas was more than 99.8%. With no significant mutations, the sequence analysis suggests that the *S. suis Cps3L* gene is stable.

It is well known that serotyping of *S. suis* is crucial to gaining insights into bacterial epidemiology and pathogenicity. Disease-associated serotypes 1, 1/2, 2, 3, 4, 5, 6, 7, 8, 9, and 14 were found to be prevalent according to the study of Murray et al. [24], but different *S. suis* serotypes exhibit diverse population structure and pathogenicity characteristics. Hence, it is important to not only focus on disease-associated serotypes but also ST. In the light of MLST analysis, the main ST subtypes of SS3 are ST1801, ST15, ST966, and ST35. The *S. suis* strain YA belongs to the ST1801 subtype, which belongs to the same branch as ST54, ST16, ST15, and ST1 in the phylogenetic tree. Notably, ST16 and ST1 were commonly pathogenic ST from serotypes 2 and 9, respectively [15]. The result of animal infection experiments of the *S. suis* strain YA to mice and piglets suggested that the *S. suis* YA is a pathogenic strain. Therefore, the pathotype of the SS3 strain YA is worth studying.

The strongly virulent strains carry numerous critical genes associated with virulence, notably including *mrp*, *sly*, *IdeSsuis*, *igdE*, *sbp2*, *hp0197*, *hp0272*, *rgg*, and *tran*, which have been identified as potential zoonotic virulence factors in a recent study [25]. The *S. suis* strain YA carried the most putative virulence-associated genes among the serotype 3 strains studied in our study. At present, the focus of vaccine research is to identify the primary virulence factors responsible for disease occurrence and to analyze their immunogenicity to develop a subunit vaccine that could protect against multiple serotypes of *S. suis*. To this end, studies have shown that the pathogenicity of *S. suis* is closely related to the virulence genes present, with *sly*, *epf*, *mrp*, and *cps* serving as representatives of virulent strains. Petrocchi Rilo et al. [26] conducted serotyping and virulence gene testing on 207 strains of *S. suis* collected from 10 autonomous regions in Spain from 2019 to 2020. The results showed that the virulence gene combinations of different serotypes were similar, but not identical. The virulence gene combinations of type 1 and type 2 strains were *epf*, *mrp*, *sly*, *luxS* and *epf*, *mrp*, *sly*, *luxS*, *gapdh*, while the virulence gene combinations of type 3 and type 9 strains were *mrp*, *sly*, *luxS* and *mrp*, *sly*, *luxS*, *gapdh*, respectively. Notably, the *S. suis* strain YA exhibited a diverse array of virulence genes, including highly virulent strains of the *sly*, *mrp*, *cps*, and *epf* genes. Biologically, the strain adheres to the cell surface through the anti-phagocytotic action of virulence factor *mrp* [27]. Other studies have shown that the virulence factor *sly* could reshape the cytoskeleton of cerebral microvascular endothelial cells, which is beneficial for *S. suis* to cross the blood-brain barrier through the gap between the cerebral microvascular endothelial cells [28]. In addition, the cytotoxicity of *sly* contributed to the extracellular survival of *S. suis* [28,29], and stimulated the secretion of inflammatory factors by host cells [30]. In this study, the isolated strain YA carried the *mrp* and *sly* genes, explaining why the strain caused severe meningitis in mice and piglets. Although various virulence factors have been associated with the pathogenicity of *S. suis*, pathogenicity seems to be the result of the complex interaction between several diverse virulence factors and further in-depth studies are needed. Thus, SS3 strains belonging to ST1801 may pose a significant threat to both humans and pigs, highlighting the need for continuous monitoring.

Antibiotics are one of the most effective methods for treating *S. suis*, but the overuse of antibiotics in production has led to the evolution of multi-drug-resistant bacteria. Other studies have shown that resistance to various antibiotics has emerged in more than 40% of isolated *S. suis*, while only about 10% of strains are highly sensitive to drugs. The results of the drug resistance gene testing in this study were consistent with the results of the drug sensitivity testing. The strain contained the *tet(W)* and *erm(B)* genes and was resistant to tetracyclines (minocycline and doxycycline) and macrolides (erythromycin). The main ribosomal protective effects in Gram-positive bacteria are mediated by *tet(M)* [31] and there is a suspected genetic link between *erm(B)* and *tet(M)*, which is regarded as the primary mechanism for the spread of streptococcal bacteria resistant to both macrolide and tetracycline antimicrobials [32]. Additionally, the *S. suis* strain YA was resistant to aminoglycoside (amikacin, gentamicin, streptomycin), lincosamides, compound sulfamethoxazole (trimethoprim sulfamethoxazole), and β-lactam antibiotics (cefuroxime sodium), despite the absence of corresponding resistance genes in the strain. It was confirmed that the clinical isolate of *S. suis* from the pig farm exhibited severe multidrug-resistant behavior, supporting our findings, and highlighting the importance of monitoring the drug sensitivity of *S. suis*. However, the *S. suis* strain YA did not contain any ICEs associated with antibiotic resistance genes. This finding aligned with the results of other studies, suggesting that the predominant vehicles for the spread of antibiotic resistance genes may vary by country or region and the strains containing ICEs were mainly discovered in other countries [15]. The detection of drug resistance in clinical isolates offers timely and effective guidance for breeding farms to scientifically and reasonably choose drugs, to prevent the emergence of multidrug-resistant strains.

## 5. Conclusions

A pathogenic *S. suis* strain YA of serotype 3 was isolated from infected piglets. And the presence of the relevant virulence genes and the molecular genetic characteristics of the strain were verified and determined, respectively. These findings contribute to a better understanding of the bacterial genome and the pathogenic properties of SS3 and present a foundation for understanding the clinical manifestations of *S. suis* to establish effective therapeutic strategies.

## Figures and Tables

**Table 1:** Characteristics of various *S. suis* strains used for analysis in this study.

Strain	Accession Number	Serotype	ST	Source	Location	Year
ISU2414	CP030023.1	2	/	NCBI	USA	2014
ISU2614	CP031377.1	2	28	NCBI	USA	2014
ISU2660	CP031379.1	2	787	NCBI	USA	2014
BM407	NC_012926.1	2	1	NCBI	Vietnam	2004
HA0609	CP024126.1	2	28	NCBI	China	2006
05HAS68	CP002007.2	2	28	NCBI	China	2006
NSUI002	CP011419.1	2	28	NCBI	Canada	2008
DNC15	CP102148.1	16	/	NCBI	Denmark	2022
DNR43	CP102143.1	2	28	NCBI	Denmark	2022
DNC49	CP102140.1	2	28	NCBI	Denmark	2022
2018WUSS151	CP101844.1	8	1241	NCBI	China	2018
WUSS030	CP110141.1	8	25	NCBI	China	2017
YB51	CP006645.1	3	35	NCBI	China	2013
ST3	CP002633.1	3	35	NCBI	China	2014
INT-01	CP041994.1	3	35	NCBI	Korea	2018
MY1C3_3B	CP134487.1	3	966	NCBI	Canada	2016
PH2016-081	CP134474.1	3	15	NCBI	Germany	2016
YA	CP149804.1	3	1801	This study	China	2022
6407	CP008921.1	4	54	NCBI	Denmark	2014
NJ3	CP082203.1	9	240	NCBI	China	2005
Ss_109	CP139876.1	9	16	NCBI	Spain	2018
NLS50	CP134488.1	9	16	NCBI	Netherlands	2017
GZ0565	CP017142.1	9	243	NCBI	China	2005

## Data Availability

The data that support the findings of this study are available on reasonable request from the corresponding author.

## Supplementary Materials

The following supporting information can be downloaded at: https://www.mdpi.com/article/10.3390/pathogens14020192/s1. Table S1: Results of LD50 test for *S. suis* YA challenged mice. Table S2: The 35 putative virulence-associated genes analyzed in this study. Table S3: Background information on the strains used in the pathogenicity experiments of mice. Figure S1: Amplification of 16S rRNA and *cps 3L* gene by PCR. Figure S2: Organ disease of mice infected with *S. suis*. Note: A~F: Hart, liver, spleen, lungs, kidneys, and brain of *S. suis* challenged mice, G~L: Hart, liver, spleen, lungs, kidneys, and brain of negative control mice.

## Abbreviations

The following abbreviations are used in this manuscript:

*S. suis*	*Streptococcus suis*
SS3	*Streptococcus suis* serotype 3
CPS	capsular polysaccharide
WGS	whole genome sequencing
MLST	multilocus sequence type
PBS	phosphate-buffered saline
LD_50_	the median lethal dose
