# Supplementary material for: Isolation, Identification, and Molecular Genetic Characteristics of a Pathogenic Strain of Streptococcus suis Serotype 3
Source: Pathogens. 2025 Feb 14;14(2):192. doi: 10.3390/pathogens14020192 (PMC11858596; doi:10.3390/pathogens14020192)
Supplement: Supplementary file 1 [file pathogens-14-00192-s001.zip › pathogens-3483654-supplementary.pdf]

Supplementary materials

Table S1 Results of LD_{50} test for *S. suis* YA challenged mice.

Strain	Group	Challenge dose（CFU）	Number of Mice	Number of deaths	Death rate（%）
YA	I	5.6×10^8	10	10	100
	II	1.7×10^8		9	90
	III	5.2×10^7		8	80
	IV	1.6×10^7		7	70
	V	4.8×10^6		1	10

Table S2 The 35 putative virulence-associated genes analyzed in this study.

Genes	Annotation/ Function	Protein ID (GenBank)
**Adhesins and cell surface factors**	**Annotation**	
atl_2	Autolysin	WP_398595430
ef	Extracellular factor (EF)	ACU12361.1
enolase	Enolase	CAZ56254.1
fbps	Fibronectin-fibrinogen binding protein (FBPS)	AAS67693.1
hp0197	HP0197	CAR44487.1
hp0272	HP0272	CAR44630.1
HtpsC	Type II histidine triad protein (HtpsC)	AHN60101
mrp	Muraminidase released protein (MRP)	CAA45781.1
ofs	Serum opacity factor (OFS)	AAX56334.1
oppA	Oligopeptide-binding protein precursor (OppA)	AIC32936.1
sao	Surface antigen one (Sao)	GAW37806.1
sbp2	Putative pilin subunit (Sbp2)	BAG74779.1
srtA	Sortase A	WP_172069557.1
ss05_1311	Fibronectin-binding protein (Ssa)	CAR46398.1
**Toxins and inflammation**	**Annotation**	
abpb	Amylase-binding protein B (Abpb)	CAR46535.1
clpP	Degradative enzyme ClpP	GAW38484.1
clpX	Degradative enzyme ClpX	WNG13042.1
Hp1717	HP1717	CAR47197.1
sly	Suilysin	WNG11599.1
SspA	Surface-associated subtilisin-like protease (SspA)	CYU04486.1
**Immunoevasion**	**Annotation**	
dltA	DltA	WNG12881.1
DPP IV	Dipeptidyl peptidase IV (DPP IV)	BCK44694.1
IdeSsuis	IdeSsuis	CAR45112.1
igdE	IgdE	CAR47313.1
pgdA	PgdA	QOE30891.1
SntA	SntA	WNG12214.1
SsnA	SsnA	BCK46421.1
**Regulatory factors**	**Function**	
codY	DNA-binding repressor	WNG12583.1
gidA	Glucose-inhibited division protein (GidA)	GAW38097.1
luxS	Quorum sensing component	ACB28477.1
revS	Orphan response regulator	AAM78179.1
rgg	Transcriptional regulator	AWX96798.1
stk	Signal transduction regulation	AGM49306.1
stp1	Serine threonine phosphatase (Stp 1)	CAR44893.1
tran	Transcriptional regulator	CAR44956.1

Table S3 Background information on the strains used in the pathogenicity experiments of mice.

Strains	Serotypes	Host	Isolation Source
YA	3	Diseased Pig	Lung
PT1016	/	Diseased Pig	/
PTSF9	4	Diseased Pig	/
ND1111	29	Diseased Pig	/
ND1114	15	Diseased Pig	/
ND0827	9	Diseased Pig	Lung

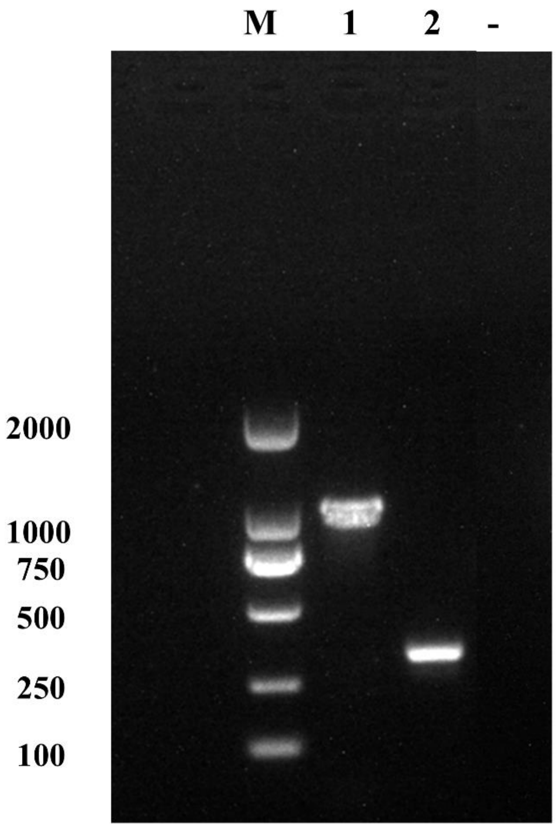

Figure S1 Amplification of 16S rRNA and *cps 3L* gene by PCR

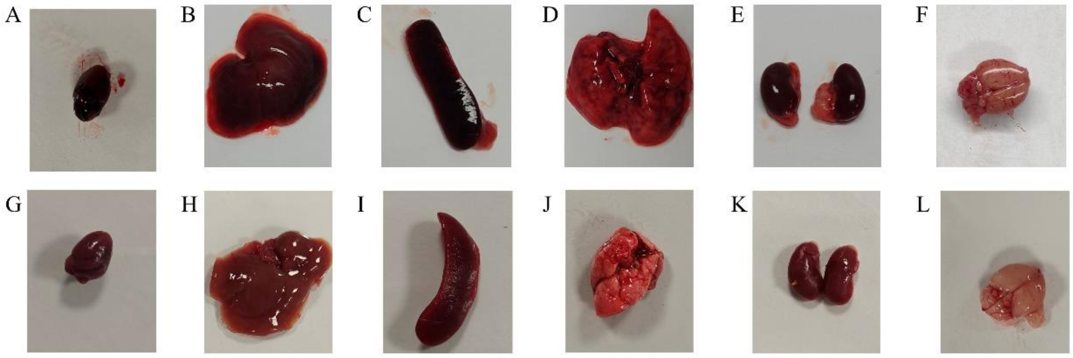

Figure S2 Organ disease of mice infected with *S. suis*.

Note: A~F: Hart, liver, spleen, lungs, kidneys, and brain of *S. suis* challenged mice, G~L: Hart, liver, spleen, lungs, kidneys, and brain of negative control mice.
